# Grey-to-white matter ratio on computed tomography for predicting neurological outcome in patients with heat stroke: a retrospective cohort study

**DOI:** 10.3389/fneur.2025.1556822

**Published:** 2025-05-13

**Authors:** Hua Wei, Hongling Zhu, Menglong Liu, Xiaodan Zhu, Anyong Yu, Can Luo, Qingbo Zeng, Fating Zhou, Haizhen Duan

**Affiliations:** ^1^Department of Emergency Medicine, Affiliated Hospital of Zunyi Medical University, Zunyi, Guizhou, China; ^2^Chongqing Key Laboratory of Emergency Medicine, Chongqing, China; ^3^Department of Interventional Radiology, Yichang Central People’s Hospital, Yichang, China; ^4^Bioengineering College, Chongqing University, Emergency department, Chongqing University Central Hospital (Chongqing Emergency Medical Center), Chongqing, China

**Keywords:** heat stroke, grey-to-white matter ratio, basal ganglia, neurological outcome, computed tomography (CT)

## Abstract

**Objective:**

Grey-to-white matter ratio (GWR) is an early and sensitive indicator of cerebral oedema in patients with hypoxic-ischaemic brain injury, we aimed to evaluate the prognostic value of GWR for predicting neurological outcome in heat stroke patients.

**Methods:**

This multicentre retrospective analysis included 86 patients with heat stroke patients who underwent cranial computed tomography (CT). Patients were stratified by Cerebral Performance Category (CPC) scores at discharge: good outcome (CPC 1–2, *n* = 65) versus poor outcome (CPC 3–5, *n* = 21) in the derivation cohort. Seven GWR parameters were calculated from Hounsfield unit measurements at three different regions (basal ganglia, centrum semiovale, high convexity): putamen/corpus callosum (PU/CC), caudate nucleus/posterior limb of internal capsule (CN/PLIC), CN/CC, PU/PLIC, GWR_basal ganglia_, GWR_cerebrum_, and GWR_average_. Prognostic performance of GWR was compared with qSOFA using receiver operating characteristic (ROC) analysis. And a validation cohort was used to verify the reliability.

**Results:**

All GWRs were significantly lower in the poor outcome group than in the good outcome group. ROC analysis showed the following areas under the curve: PU/CC, 0.836; CN/PLIC, 0.815; CN/CC, 0.858; PU/PLIC, 0.814; GWR_basal ganglia_, 0.855; GWR_cerebrum_, 0.803; GWR_average_, 0.837. The cutoff values with 90.77% specificity in predicting poor outcome were as follows: PU/CC, 1.20 (sensitivity, 76.19%); CN/PLIC, 1.17 (sensitivity, 52.38%); CN/CC, 1.20 (sensitivity, 76.19%); PU/PLIC, 1.20 (sensitivity, 61.90%); GWR_basal ganglia_, 1.23 (sensitivity, 80.95%); GWR_cerebrum_, 1.19 (sensitivity, 57.14%); GWR_average_, 1.23 (sensitivity, 71.43%). The sensitivity of GWR_basal ganglia_ significantly increased when combined with qSOFA in the derivation and validation cohorts.

**Discussion:**

A low GWR was strongly associated with poor outcome in the heat stroke patients. The GWR may be useful as an objective early predictor of poor neurological outcome in the heat stroke patients. Incorporating the GWR with qSOFA significantly enhanced the prediction performance.

## 1 Introduction

Heat stroke, which is caused by global warming and the increasing intensity of global heatwaves, is a common and life-threatening disorder with a high mortality rate (1). Approximately 1.2 billion people would be at risk of a heat stroke worldwide annually by the year 2,100, and the case fatality rate of heat stroke is 10–20% (2, 3). For patients with severe heat stroke, the 28-day mortality rate is nearly 60% (4, 5). Furthermore, numerous survivors have long-term neurological sequelae, such as dysarthria, cognitive impairment, personality change, and limb paresis (3, 6). Brain imaging of survivors with neurological dysfunction identified damage to the prefrontal cortex, cerebellum, and/or hippocampus several months later (7). Thus, early and accurate assessment of neurological outcome is vital in making appropriate therapeutic decisions in patients with heat stroke.

Currently, several classic indicators for evaluating prognosis of heat stroke have been identified: temperature, heart rate, systolic blood pressure, creatinine, aspartate aminotransferase, activated partial thromboplastin time, international normalised ratio, and cooling time (8–10). Based on these indicators, predictive prognosis systems for heat stroke have been developed, including the Sequential Organ Failure Assessment (SOFA) score, Acute Physiology and Chronic Health II score, and Exertional Heat Stroke Score (11, 12). However, all above scoring systems cannot be rapidly obtained due to the requirement for several tests. Thus, a novel, easy-to-access and reproducible tool is needed for predicting neurological outcome of heat stroke patients.

During diagnostic procedures, cranial computed tomography (CT) scans are performed to rule out stroke and brain haemorrhage in patients with heat stroke. Simultaneously, brain oedema can be assessed by differences in the grey and white matter in cranial CT (13, 14). Grey matter (GM) is composed of neuronal bodies and synapses; white matter (WM) mainly consists of myelinated axons. The differences between GM and WM on cranial CT images arise because of the low lipid content and high-water content of GM resulting in a lower carbon concentration as well as a higher oxygen concentration, increasing the level of photoelectric uptake (15). The selective susceptibility of GM to ischemia is due to its higher metabolic rate, greater blood flow, and susceptibility to excitotoxicity (15). A previous retrospective study revealed that severe loss of grey–white matter discrimination is an early and sensitive radiographic indicator of severe brain damage in patients with heat stroke (13). The loss of grey-white matter discrimination can be measured and quantified by the ratio of the grey matter to the white matter (GWR), which is a recommended and effective tool for predicting neurological outcome in comatose cardiac arrest survivors by guidelines for cardiopulmonary resuscitation (14, 16). Based on current evidence, we aimed to evaluate the reliability of GWR in predicting neurological prognostication for patients with heat stroke.

## 2 Materials and methods

### 2.1 Ethics approval

The study was approved by the Human Ethical Committee of Chongqing Emergency Medical Center and was in accordance with the Declaration of Helsinki. The Ethics Committee/Institutional Review Board waived the requirement for written informed consent to participate owing to the retrospective nature of the study, but the patients provided informed consent for the publication of the cranial CT images. All clinical information about the patients was maintained in confidence, and the data were analysed in an anonymous manner.

### 2.2 Study population

This multicentre retrospective study enrolled heat stroke patients from the Affiliated Hospital of Zunyi Medical University, Chongqing Emergency Medicine Hospital, Fifth People’s Hospital of Chongqing, Dianjun District People’s Hospital of Yichang and Yichang Central People’s Hospital between January 2020 and November 2023 (ChiCTR2400079671). Cases of heat stroke were screened using the International Classification of Diseases, Tenth Revision code from the electronic database. Heat stroke was defined as a core body temperature >40°C, accompanied by central nervous system abnormalities, including coma, delirium, and convulsion (17). Clinical data, including age, sex, comorbidity, temperature, presentation, laboratory tests, cranial CT, and outcome, were collected from medical records. The qSOFA score of enrolled patients on admission were obtained.

**Figure 1:**
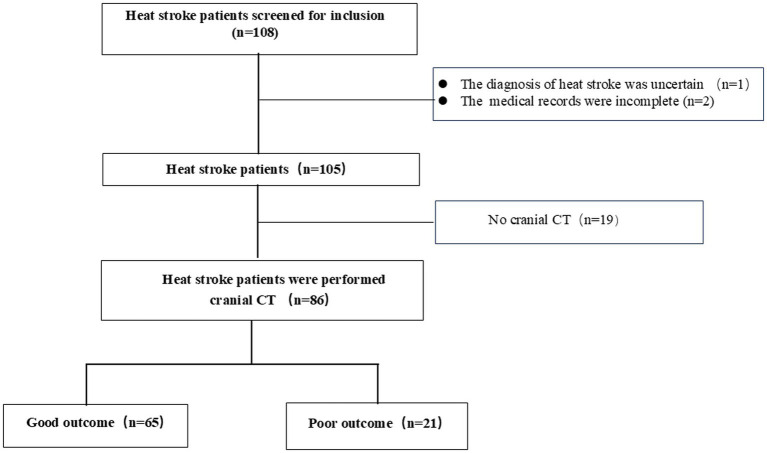
Flow diagram illustrating heat stroke patients’ selection process.

The inclusion criteria were patients who met the heat stroke diagnostic criteria, who were older than 18 years, and who underwent cranial CT. Those with incomplete medical records and data, with traumatic brain injury and acute stroke, and who underwent cranial CT after resuscitation were excluded. The reasons for performing cranial CT scans were not relevant to this study. Most of the patients with heat stroke underwent cranial CT to rule out primary intracranial events. Heat stroke patients were divided into derivation and validation groups according to the city. These patients were included in the derivation group from the Zunyi and Chongqing. The remaining heat stroke patients from Yichang were included in the validation group. Of the 108 patients with heat stroke who were enrolled into derivation cohort, 22 were excluded; finally, 86 patients with heat stroke were included in the derivation group (Figure 1). In addition, 42 heat stroke patients from Yichang were used to verify the reliability of GWR.

### 2.3 GWR determination

**Figure 2:**
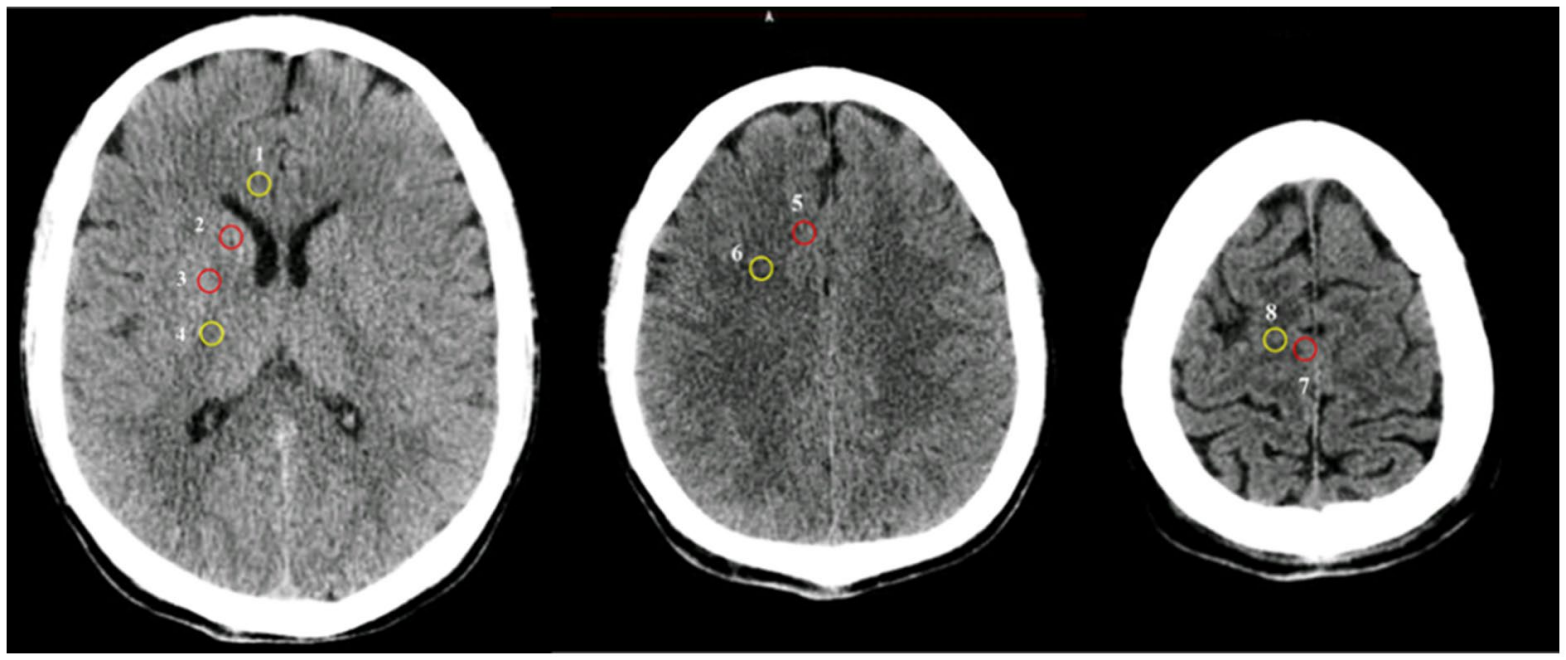
Circular regions of interest were placed bilaterally in the Cranial CT. 1 corpus callosum (CC), 2 caudate nucleus (CN), 3 putamen (PU), 4 posterior limb of internal capsule (PLIC), 5 cortex matter at the centrum semiovale level (MC1), 6 white matters at the centrum semiovale level (WM1), 7 cortexes at the high convexity level (MC2), 8 white matters at the high convexity level (WM2). Red circle: grey matter, yellow circle: white matter.

Participants were scanned by a SOMATOM Sensation 64 CT scanner (Siemens Healthiness, Erlangen, Germany) with 5-mm slices. Regions of interest (ROI) were detected independently by three investigators. They were blinded to the outcome and clinical information of patients during GWR determination (17). After adjustment of the window to the brain, investigators reviewed CT scans using a commercial image-viewing software and identified comparable brain slices. Circular regions of measurement (10 mm^2^) were placed over the ROI bilaterally (Figure 2), and the average attenuation was recorded with Hounsfield units (HU). The basal ganglia level was determined from the putamen (PU), caudate nucleus (CN), corpus callosum (CC), and posterior limb of internal capsule (PLIC). The centrum semiovale and high convexity levels were determined from the medial cortex (MC1 and MC2) and medial white matter (MW1 and MW2), respectively. The GWRs were calculated by seven methods according to previous reports (18, 19): PU/CC, CN/PLIC, CN/CC, PU/PLIC, GWR_basal ganglia_ = (PU + CN)/(CC + PLIC), GWR_cerebrum_ = (MC1 + MC2)/(WM1 + WM2), and GWR_average_ = (PU + CN + MC1 + MC2)/(CC + PLIC+WM1 + WM2).

### 2.4 Outcome measure

The primary outcome of patients with heat stroke was clinical outcome at hospital discharge, which was assessed using the Cerebral Performance Category (CPC) score (Supplementary Table S1), and patients were divided into good outcome group (CPC 1–2) and poor outcome group (CPC 3–5).

### 2.5 Statistical analyses

Continuous variables are expressed as mean±standard deviation or median with interquartile ranges. Categorical data are expressed as number and frequency. Differences between two groups were tested with the independent two-sample *t* test or Mann–Whitney U test. Comparisons of categorical variables were tested using the chi-square test or Fisher’s exact test, as appropriate. Obtaining the optimal threshold for predicting prognosis with GWRs was determined through receiver operating characteristic (ROC) curve analysis. The statistical performance of the outcome predictive models was estimated by the area under the curve (AUC), with 95% confidence interval (CI). These AUC values were compared with the Delong test. All statistical analyses were performed in SPSS version 19.0 (IBM Corp., Armonk, NY, United States). A two-tailed *p* value <0.05 was considered statistically significant.

## 3 Results

### 3.1 Clinical characteristics

The average age of the patients was >65 years, most were male, and most presented with underlying diseases, including hypertension, diabetes, and coronary artery disease. Of the 86 patients, 65 had good neurological outcome, and 21 had poor neurological outcome. The baseline characteristics are presented in Table 1. Except for faecal or urinary incontinence, there was no significant difference between the groups in terms of age, sex, comorbidities, symptoms, and duration from onset of symptoms to cranial CT scans. However, patients in the poor outcome group had higher rectal temperature (41.8°C versus 40.5°C), heart rate (123.0 versus 97.0 bpm), respiratory rate (28.0 versus 20.0 bpm), qSOFA (3.0 versus 1.0), and length of stay of hospital (12.0 versus 5.0 days) than those in good outcome group. Furthermore, the patients in the poor outcome group were more likely to experience multiorgan dysfunction (95.2% versus 36.9%) and to be admitted to the intensive care unit (95.2% versus 38.5%) than those in the good outcome group.

**Table 1:** Characteristics of the study population (*n* = 86).

	Good outcome (*n* = 65)	Poor outcome (*n* = 21)	*p*-value
Age (years)	70.0 (56.5–77.5)	77.8 (55.5–85.5)	0.231
Male gender, *n* (%)	36 (55.4)	15 (71.4)	0.193
Comorbidities			
Hypertension, *n* (%)	23 (35.4)	5 (23.8)	0.325
Diabetes, *n* (%)	11 (16.9)	4 (19.0)	0.527
Coronary artery disease, *n* (%)	3 (4.6)	2 (9.5)	0.592
Stroke, *n* (%)	3 (4.6)	3 (14.3)	0.153
Symptoms and signs			
Fecal or urinary incontinence, *n* (%)	4 (6.1)	6 (28.6)	0.012
Cramp, *n* (%)	13 (20)	3 (14.3)	0.751
Weakness, *n* (%)	13 (20)	3 (14.3)	0.751
Vomiting, *n* (%)	5 (7.7)	2 (9.5)	1.000
From onset of symptoms to admission (h)	2.0 (1.00–5.00)	3.0 (1.50–6.50)	0.430
From onset of symptoms to cranial CT	2.8 (1.65–5.45)	6 (1.95–23.00)	0.094
Rectal temperature (°C)	40.5 (40.1–40.4)	41.8 (40.4–42.2)	0.022
Heart rate (bpm)	97.0 (82.5–115.5)	123.0 (98.5–142.0)	0.002
Respiratory rate (bpm)	20.0 (19.0–24.0)	28.0 (23.5–36.0)	<0.0001
Shock, *n* (%)	4 (6.2%)	10 (47.6%)	<0.0001
MODS, *n* (%)	24 (36.9%)	20 (95.2%)	<0.0001
qSOFA scores	1.0 (0.0–2.0)	3.0 (2.0–3.0)	<0.0001
Staying intensive unit, *n* (%)	25 (38.5%)	20 (95.2%)	<0.0001
Length of stay (days)	5.0 (2.0–9.0)	12.0 (2.0–24.5)	0.034

CT, computed tomography; MODS, multiple organ dysfunction syndrome; qSOFA, quick sepsis related organ failure assessment.

### 3.2 Cranial CT finding

**Figure 3:**
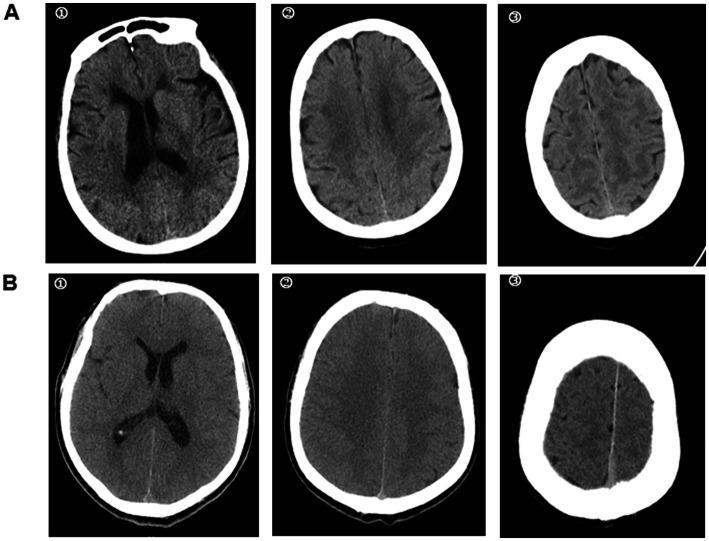
Cranial CT in heat stroke patients with different neurological outcome. A heat stroke patient was admitted to the hospital. Cranial CT in the emergency department showed a well-defined grey-white matter difference in the brain. He was discharged from the hospital with CPC 1 **(A)**. While, the cranial CT in another heat stroke patient suggested cerebral oedema with loss of grey-white matter discrepancy. The CPC was 5 at discharge from hospital **(B)**. ① basal ganglia level; ② centrum semiovale level; ③ high convexity level.

Cranial CT indicated cerebral sulci and effacement of brainstem cisterns, decreased cortical density, and loss of the normal differentiation of the white and the grey matter. The imaging signs were clearly observed in the basal ganglia, centrum semioval, and high convexity levels. For patients with poor outcome, diffuse cerebral oedema was clearly visible, and the values of the white matter were nearly similar to those of the grey matter (Figure 3). There were no cases of central nervous system haemorrhage or displaced anatomical structures.

### 3.3 GWR on cranial CT

The attenuation values and GWRs are presented in Table 2. The attenuation values of the grey matter at high convexity were significantly lower in the poor outcome group than in the good outcome group, and the attenuation values of the white matter at the basal ganglia was higher in poor outcome group, whereas the grey matter attenuation values of basal ganglia and centrum semiovale showed no significant difference between the two groups. Interestingly, both white and grey matter attenuation values failed to show significant differences at the centrum semiovale level. All seven GWRs were significantly lower in the poor outcome group than in the good outcome group: median CN/CC: poor outcome group, 1.110; good outcome group, 1.364 (*p* < 0.05); median PU/CC, poor outcome group, 1.097; good outcome group, 1.350 (*p* < 0.05); GWR_basal ganglia_: poor outcome group, 1.136; good outcome group, 1.372 (*p* < 0.05).

**Table 2:** Attenuation values and grey to white matter ratios.

	Good outcome (*n* = 65)	Poor outcome (*n* = 21)	*p-*value
Basal ganglia			
Caudate nucleus (CN)	31.0 (28.0–33.0)	30.0 (27.5–32.0)	0.269
Putamen (PU)	31.0 (28.0–33.0)	30.0 (28.0–33.0)	0.793
Corpus callosum (CC)	23.0 (20.0–25.0)	27.0 (24.0–28.5)	<0.0001
Posterior limb of internal capsule (PLIC)	22.0 (20.0–24.0)	26.0 (23.5–26.5)	0.001
Centrum semiovale			
Medial cortex (MC1)	29.0 (26.0–32.5)	27.0 (24.0–31.5)	0.117
Medial white matter (MW1)	21.0 (19.0–24.1)	23.0 (22.5–24.0)	0.103
High convexity			
Medial cortex (MC2)	29 (26.0–32.0)	27.0 (27.0–29.5)	0.043
Medial white matter (MW2)	21.0 (19.0–25.0)	22.0 (21.0–26.0)	0.111
Grey matter to white matter ratio (GWR)			
PU/CC	1.350 (1.280–1.450)	1.097 (1.037–1.242)	<0.0001
CN/PLIC	1.391 (1.293–1.523)	1.154 (1.113–1.275)	<0.0001
CN/CC	1.364 (1.250–1.461)	1.100 (1.052–1.244)	<0.0001
PU/PLIC	1.391 (1.275–1.477)	1.154 (1.108–1.307)	<0.0001
GWR_basal ganglia_	1.372 (1.307–1.452)	1.136 (1.094–1.221)	<0.0001
GWR_cerebrum_	1.353 (1.292–1.418)	1.163 (1.077–1.272)	0.001
GWR_average_	1.2321 (1.262–1.405)	1.134 (1.115–1.305)	<0.0001

CC, corpus callosum; CN, caudate nucleus; PU, putamen; PLIC, posterior limb of internal capsule; MC1, cortex matter at the centrum semiovale level; WM1, white matter at the centrum semiovale level; MC2, cortexes at the high convexity level; WM2, white matter at the high convexity level; GWR, grey white matter ratio.

### 3.4 Prognostic performances of GWRs

**Figure 4:**
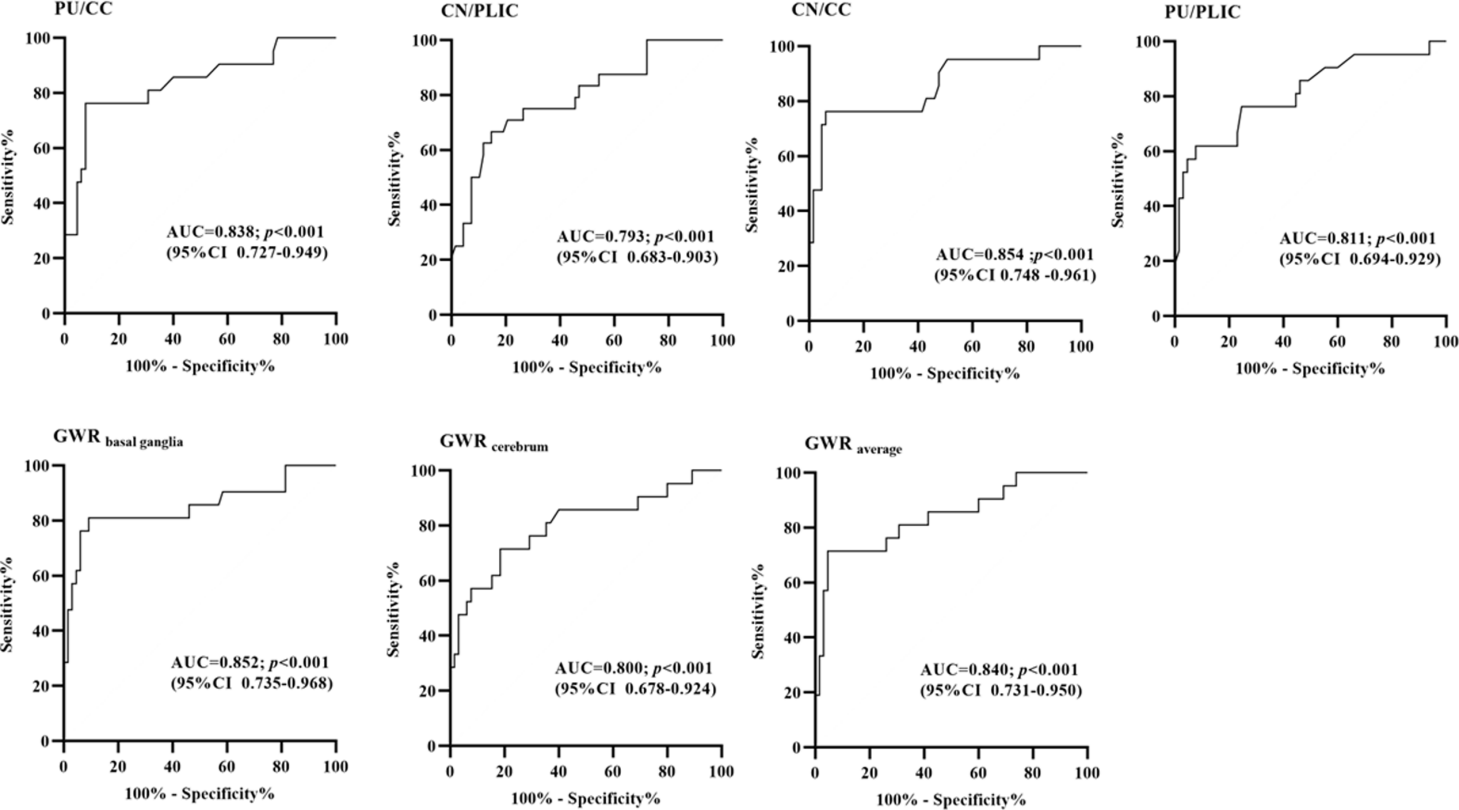
Receiver-operating characteristic curves for 7 different GWRs with multivariate logistic regression (AUC, area under curve; CI, confidence intervals).

For the ROC curve analysis for the prediction of poor outcome (Figure 4; Table 3), all seven GWRs predicted poor outcomes, with sensitivities ranging from 19.05 to 28.57% at cut-off values with 100% specificity. The AUC values of the GWRs were between 0.793 and 0.854. The CN/CC had an AUC of 0.854 (95% CI, 0.748–0.961), and its cut-off value for 100% specificity of predicting the poor outcome was 1.06. GWR_basal ganglia_ had an AUC of 0.852 (95% CI, 0.735–0.968), and its cut-off value for 100% specificity for poor outcome was 1.08. At 90.77% specificity, GWR_basal ganglia_ had the highest sensitivity (80.95%; cut-off value, 1.21) among all methods.

**Table 3:** Sensitivity and specificity for poor outcome of attenuation measurements and GWR.

	Cut-off value	Sensitivity	Specificity	PPV	NPV	AUC (95% CI)
PU/CC	1.04	28.57%	100%	100%	58.33%	0.838
	1.18	76.19%	90.77%	89.19%	79.22%	(0.727–0.949)
CN/PLIC	1.10	20.83%	100%	100%	58.24%	0.793
	1.15	50.00%	90.77%	85.01%	64.58%	(0.683–0.903)
CN/CC	1.06	28.57%	100%	100%	58.33%	0.854
	1.18	76.19%	90.77%	90.83%	79.50%	(0.748–0.961)
PU/PLIC	1.08	19.05%	100%	100%	55.26%	0.811
	1.18	61.90%	90.77%	87.02%	70.43%	(0.694–0.929)
GWR_basal ganglia_	1.08	28.57%	100%	100%	58.33%	0.852
	1.21	80.95%	90.77%	89.76%	81.91%	(0.735–0.968)
GWR_cerebrum_	1.08	28.57%	100%	100%	58.33%	0.800
	1.17	57.14%	90.77%	86.09%	67.93%	(0.67–0.924)
GWR_average_	1.10	19.05%	100%	100%	55.26%	0.840
	1.20	71.43%	90.77%	88.56%	76.06%	(0.731–0.950)

### 3.5 qSOFA improves GWR for predicting poor outcome

**Figure 5:**
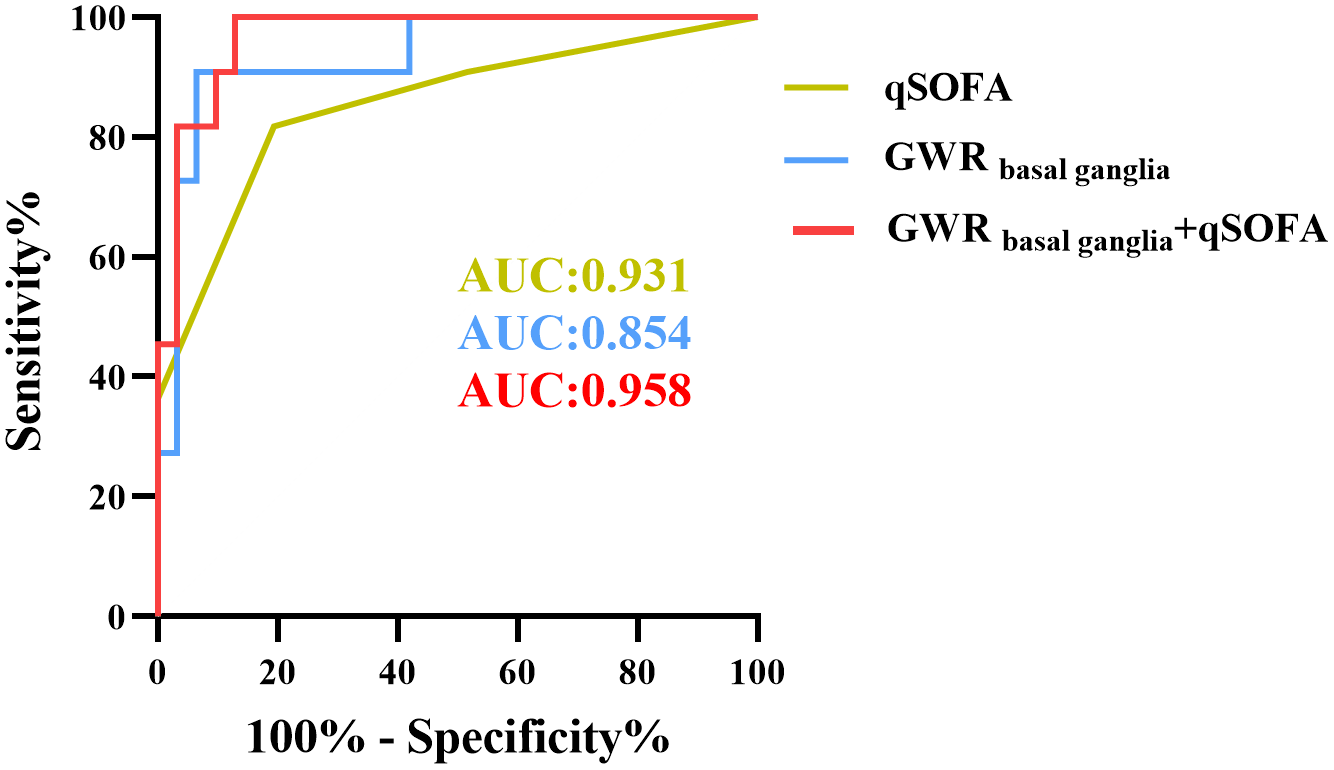
GWR improved qSOFA for predicting neurological outcome in heat stroke patients.

As described before, qSOFA is a reliable predictor in assessing outcome of heat stroke. Compared to the SOFA score, it consists of three parameters and not requiring auxiliary examinations. Our previous results also revealed that patients with heat stroke of poor outcome presented with significantly higher qSOFA scores than those in the good outcome group. Further analysis indicated that qSOFA had an AUC of 0.931 (95% CI, 0.878–0.984), and its cutoff value for 67.69% specificity for poor outcome was 2, but the specificity increased to 70.77% when combined with GWR_basal ganglia_ (Figure 5), and the sensitivity of GWR_basal ganglia_ with qSOFA increased to 61.90%. Compared to the AUC predicting neurological prognosis with GWR_basal ganglia_, the AUC was significantly greater after combination of qSOFA score (*p* = 0.034 < 0.05) with the Delong tests.

### 3.6 Validation of the GWR in predicting neurological outcome in heat stroke patients

**Figure 6:**
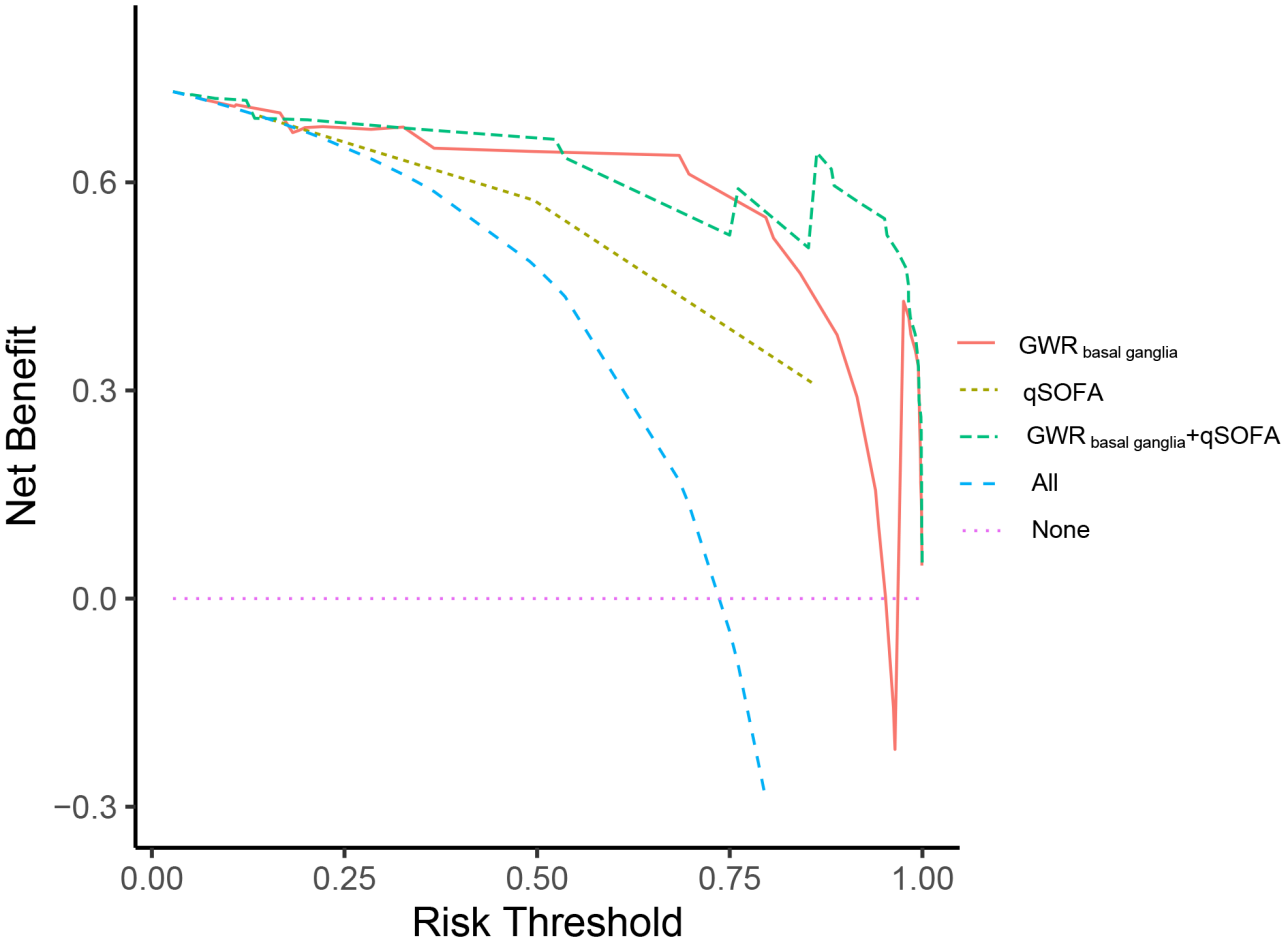
Decision-curve analysis (DCA) for predicting neurological outcome of heat stroke patients at discharge. Decision curve analysis for the qSOFA, GWR _basal ganglia_ and GWR _basal ganglia_ + qSOFA. The x-axis displayed the threshold probability, and y-axis detected the net benefit. Red line: GWR _basal ganglia;_ yellow line: qSOFA; green line: GWR _basal ganglia_ +qSOFA.

To confirm the clinical usefulness of GWR, we collected an additional 42 heat stroke patients in the validation cohort. The median age was 69.0 years, and this group included 26 male patients (61.9%). Of 42 heat stroke patients, 11 patients (26.19%) presented with poor outcome at discharge. Furthermore, we detected the predict performance of GWR_basal ganglia_ in predicting neurological outcome. The results indicated that GWR_basal ganglia_ had AUC of 0.936 (95% CI, 0.851–1.000), and its cutoff value for 80.65% specificity for poor outcome was 1.224, but the specificity of GWR_basal ganglia_ with qSOFA increased to 90.32% (Supplementary Table S2). Combination GWR_basal ganglia_ with qSOFA was presented with greater net benefit than GWR _basal ganglia_ over a wide range of threshold probabilities (Figure 6).

## 4 Discussion

The brain is one of the organs most vulnerable to hyperthermia (20). Neurological impairments are the most characteristic clinical manifestations in patients with heat stroke (21), and these neurological symptoms may present in the early stage, may persist for a long time, and are closely related to long-term cognitive and motor disability in survivors of heat stroke. Therefore, developing a novel and effective biomarker that detects brain injury and predicts delayed central nervous system damage is important.

Currently, several neurobiomarkers, including neuron-specific enolase (NSE), S-100 calcium-binding protein B (S-100 B), glial fibrillary acidic protein, and tau protein, are known to significantly increase in patients with heat stroke (21, 22). Of these markers, NSE and S-100 B have been proposed for heat stroke encephalopathy (22, 23). Under physiological conditions, S-100 B and NSE are abundantly expressed in astrocytes and neurons, with low levels in serum and cerebrospinal fluid (24), but their concentrations increase considerably during acute brain injuries, such as traumatic brain injury, cardiac arrest, and stroke (25–27). Increasing evidence showed that the concentration of S-100 B was strongly correlated with neurological outcomes for up to 7 days post-heat stroke (9). Chun et al. (23) reported that the serum S-100 B concentration of patients with heat stroke was 5 times higher in the poor outcome group than in the good outcome group, and its sensitivity in predicting poor outcome was 86% at cutoff value of 0.61 μg/L, with 86% specificity (23). On the contrary, a study of moderate-intensity exercise with heat strain revealed no differences in serum S-100 B level during exercise (28). Limited by the scarcity of studies, the reliability of neurobiomarkers in predicting neurological prognosis remains to be further clarified.

Apart from neurobiomarkers, cranial CT is commonly used for early detection and differential diagnosis of patients with cerebrovascular accidents and those with heat stroke with impaired consciousness. Several case reports on heat stroke revealed that diffusive cerebral oedema appeared as a loss of grey–white matter discrimination, which predicts poor outcome (29, 30). In physiological state, the difference between grey and white matter is clearly visible in cranial CT (31), but this difference gradually disappears during cerebral oedema (32). This cranial CT finding is also known as “loss of boundary” or “reverse sign” and can be measured quantitatively using the GWR value (13, 19). Similarly, a lower GWR is associated with severe cerebral oedema and neurological impairments.

The present study found that the GWR of patients with heat stroke was lower in the poor outcome group than that in the good outcome group. In fact, GWR was a classic indicator of predicting neurological prognosis in patients post-cardiac arrest syndrome. The sensitivity of GWR can be affected by various factors, including ROI for determining GWR and cutoff values. A study of out-of-hospital cardiac arrest conducted by Lee et al. (33) revealed that the sensitivities of the GWR of PU/CC, PU/PLIC, CC/PLIC, and GWR_basal ganglia_ were significantly different in predicting poor outcome. Similarly, Ali et al. (34) demonstrated that GWR had good correlation with cognitive function and quality of life in the aneurysmal subarachnoid hemorrhage patients, and a low GWR indicated cognitive dysfunction. Based on the above findings, we evaluated the neurological outcome of patients with heat stroke with seven different GWRs at the basal ganglia, centrum semiovale, and high convexity levels. The ROC curve analysis revealed that GWR_basal ganglia_ presented with the highest sensitivity.

APACHE II and SOFA scores are the common tools used for predicting mortality in the emergency department. In comparison with this two scores, qSOFA can be obtained rapidly at the bedside from respiratory rate, systolic blood pressure and state of consciousness and is not reliant on arterial serum analysis, routine blood examination and coagulation tests. Although qSOFA includes consciousness, it mainly focuses on systemic dysfunction. The state of consciousness is susceptible to hypothermia treatment. Thus, the specificity for assessing the neurological outcome of heat stroke is limited. In contrast to qSOFA, GWR measures brain oedema and directly reflects brain dysfunction. Unlike qSOFA, GWR is used as an indicator of cerebral oedema. Therefore, when qSOFA is used in combination with GWR_basal ganglia_, the reliability is significantly improved.

The study has some limitations. Firstly, this was a retrospective multicentre study with a limited number of patients and quality of data. Some patients with heat stroke underwent cranial magnetic resonance imaging without CT scans. Because of the small sample number, the study might not have enrolled rare cases with favourable neurological outcome despite the development of brain oedema in the early stage. We also cannot perform subgroup analyses of heat stroke patients according to CT scanners Secondly, our hospital is the largest emergency centre in Southwest China. Patients with heat stroke are usually treated with cooling therapy out of hospital. Some of the patients returned home without hospitalisation after their temperature quickly returned to normal and neurological function improved, but inpatients are likely to have a more severe condition than patients with heat stroke in other hospitals. Thirdly, this study did not use serial cranial CT or automated GWR determination. Further studies are needed to identify the optimal time to capture CT scans for GWR determination. Fourthly, grey and white matter detection is influenced by traumatic brain injury and acute cerebral infarction, and the GWR is also disturbed in patients following cardiopulmonary resuscitation for heat stroke; therefore, the above patients were excluded from the present study. Finally, we did not evaluate neurological prognosis together with other prognostic indicators such as S-100 B and NSE. These neurobiomarkers are seldom examined in patients with heat stroke, especially in primary hospitals.

In conclusion, among patients with heat stroke who underwent cranial CT, GWR_basal ganglia_ <1.22 was a predictor of poor neurological outcome. Incorporating the GWR with qSOFA significantly improved the reliability of prediction.

## Data Availability

The original contributions presented in the study are included in the article/Supplementary material, further inquiries can be directed to the corresponding authors.

## References

[1] Patz JA Campbell-Lendrum D Holloway T Foley JA. Impact of regional climate change on human health. Nature. (2005) 438:310–7. doi: 10.1038/nature04188, PMID: 16292302

[2] Glaser J Lemery J Rajagopalan B Diaz HF Garcia-Trabanino R Taduri G. Climate change and the emergent epidemic of CKD from heat stress in rural communities: the case for heat stress nephropathy. Clin J Am Soc Nephrol. (2016) 11:1472–83. doi: 10.2215/CJN.13841215, PMID: 27151892 PMC4974898

[3] Zhong L Wu M Liu Z Liu Y Ren G Su L. Risk factors for the 90-day prognosis of severe heat stroke: a case-control study. Shock. (2021) 55:61–6. doi: 10.1097/SHK.0000000000001589, PMID: 32590693

[4] Argaud L Ferry T Le QH Marfisi A Ciorba D Achache P. Short- and long-term outcomes of heatstroke following the 2003 heat wave in Lyon, France. Arch Intern Med. (2007) 167:2177–83. doi: 10.1001/archinte.167.20.ioi70147, PMID: 17698677

[5] Hifumi T Kondo Y Shimizu K Miyake Y. Heat stroke. J Intensive Care. (2018) 6:30. doi: 10.1186/s40560-018-0298-4, PMID: 29850022 PMC5964884

[6] Garcia CK Renteria LI Leite-Santos G Leon LR Laitano O. Exertional heat stroke: pathophysiology and risk factors. BMJ Med. (2022) 1:e000239. doi: 10.1136/bmjmed-2022-000239, PMID: 36936589 PMC9978764

[7] Leon LR Bouchama A. Heat stroke. Compr Physiol. (2015) 5:611–47. doi: 10.1002/cphy.c140017 25880507

[8] Xing L Liu SY Mao HD Zhou KG Song Q Cao QM. The prognostic value of routine coagulation tests for patients with heat stroke. Am J Emerg Med. (2021) 44:366–72. doi: 10.1016/j.ajem.2020.04.062, PMID: 32389399

[9] Schlader ZJ Davis MS Bouchama A. Biomarkers of heatstroke-induced organ injury and repair. Exp Physiol. (2022) 107:1159–71. doi: 10.1113/EP090142, PMID: 35654394 PMC9529995

[10] Liu S Xing L Wang J Xin T Mao H Zhao J. The relationship between 24-hour indicators and mortality in patients with exertional heat stroke. Endocr Metab Immune Disord Drug Targets. (2022) 22:241–6. doi: 10.2174/1871530321666210122153249, PMID: 33480352

[11] Zhong L Wu M Ji J Liu Z. Usefulness of sequential organ failure assessment score on admission to predict the 90-day mortality in patients with exertional heatstroke: an over 10-year intensive care survey. Am J Emerg Med. (2022) 61:56–60. doi: 10.1016/j.ajem.2022.08.042, PMID: 36049393

[12] Li P Yang L Liu R Chen RL. The value of the exertional heat stroke score for the prognosis of patients with exertional heat stroke. Am J Emerg Med. (2021) 50:352–5. doi: 10.1016/j.ajem.2021.08.036, PMID: 34454398

[13] Szold O Reider G II Ben Abraham R Aviram G Segev Y Biderman P. Gray-white matter discrimination--a possible marker for brain damage in heat stroke? Eur J Radiol. (2002) 43:1–5. doi: 10.1016/s0720-048x(01)00467-3 12065113

[14] Panchal AR Bartos JA Cabanas JG Donnino MW Drennan IR Hirsch KG. Part 3: adult basic and advanced life support: 2020 American Heart Association guidelines for cardiopulmonary resuscitation and emergency cardiovascular care. Circulation. (2020) 142:S366–468. doi: 10.1161/CIR.0000000000000916, PMID: 33081529

[15] Gutierrez LG Rovira A Portela LA Leite Cda C Lucato LT. CT and MR in non-neonatal hypoxic-ischemic encephalopathy: radiological findings with pathophysiological correlations. Neuroradiology. (2010) 52:949–76. doi: 10.1007/s00234-010-0728-z, PMID: 20585768

[16] Nolan JP Sandroni C Bottiger BW Cariou A Cronberg T Friberg H. European resuscitation council and European Society of Intensive Care Medicine Guidelines 2021: post-resuscitation care. Resuscitation. (2021) 161:220–69. doi: 10.1016/j.resuscitation.2021.02.012, PMID: 33773827

[17] Lipman GS Gaudio FG Eifling KP Ellis MA Otten EM Grissom CK. Wilderness medical society clinical practice guidelines for the prevention and treatment of heat illness: 2019 update. Wilderness Environ Med. (2019) 30:S33–46. doi: 10.1016/j.wem.2018.10.004, PMID: 31221601

[18] Lee YH Oh YT Ahn HC Kim HS Han SJ Lee JJ. The prognostic value of the grey-to-white matter ratio in cardiac arrest patients treated with extracorporeal membrane oxygenation. Resuscitation. (2016) 99:50–5. doi: 10.1016/j.resuscitation.2015.11.009 26690647

[19] Zhou F Wang H Jian M Wang Z He Y Duan H. Gray-white matter ratio at the level of the basal ganglia as a predictor of neurologic outcomes in cardiac arrest survivors: a literature review. Front Med (Lausanne). (2022) 9:847089. doi: 10.3389/fmed.2022.847089, PMID: 35372375 PMC8967346

[20] Bazille C Megarbane B Bensimhon D Lavergne-Slove A Baglin AC Loirat P. Brain damage after heat stroke. J Neuropathol Exp Neurol. (2005) 64:970–5. doi: 10.1097/01.jnen.0000186924.88333.0d, PMID: 16254491

[21] Yaqub B Al DS. Heat strokes: aetiopathogenesis, neurological characteristics, treatment and outcome. J Neurol Sci. (1998) 156:144–51. doi: 10.1016/s0022-510x(98)00037-9, PMID: 9588849

[22] Stacey MJ Leckie T Fitzpatrick D Hodgson L Barden A Jenkins R. Neurobiomarker and body temperature responses to recreational marathon running. J Sci Med Sport. (2023) 26:566–73. doi: 10.1016/j.jsams.2023.09.011 37777396

[23] Chun JK Choi S Kim HH Yang HW Kim CS. Predictors of poor prognosis in patients with heat stroke. Clin Exp Emerg Med. (2019) 6:345–50. doi: 10.15441/ceem.18.081, PMID: 31910506 PMC6952628

[24] Wang CH Chang WT Su KI Huang CH Tsai MS Chou E. Neuroprognostic accuracy of blood biomarkers for post-cardiac arrest patients: a systematic review and meta-analysis. Resuscitation. (2020) 148:108–17. doi: 10.1016/j.resuscitation.2020.01.006, PMID: 31978453

[25] Olivecrona M Rodling-Wahlstrom M Naredi S Koskinen LO. S-100B and neuron specific enolase are poor outcome predictors in severe traumatic brain injury treated by an intracranial pressure targeted therapy. J Neurol Neurosurg Psychiatry. (2009) 80:1241–8. doi: 10.1136/jnnp.2008.158196, PMID: 19602473

[26] Honegger T Schweizer J Bicvic A Westphal LP Schutz V Inauen C. Serum S-100B adds incremental value for the prediction of symptomatic intracranial hemorrhage and brain edema after acute ischemic stroke. Eur Stroke J. (2023) 8:309–19. doi: 10.1177/23969873221145391, PMID: 37021149 PMC10068408

[27] Perkins GD Callaway CW Haywood K Neumar RW Lilja G Rowland MJ. Brain injury after cardiac arrest. Lancet. (2021) 398:1269–78. doi: 10.1016/S0140-6736(21)00953-3 34454687

[28] Cheuvront SN Chinevere TD Ely BR Kenefick RW Goodman DA McClung JP. Serum S-100beta response to exercise-heat strain before and after acclimation. Med Sci Sports Exerc. (2008) 40:1477–82. doi: 10.1249/MSS.0b013e31816d65a5, PMID: 18614943

[29] Lee S Lee SH. Exertional heat stroke with reversible severe cerebral edema. Clin Exp Emerg Med. (2021) 8:242–5. doi: 10.15441/ceem.19.085, PMID: 34649413 PMC8517459

[30] Mozzini C Xotta G Garbin U Fratta Pasini AM Cominacini L. Non-exertional heatstroke: a case report and review of the literature. Am J Case Rep. (2017) 18:1058–65. doi: 10.12659/ajcr.905701, PMID: 28974669 PMC5637572

[31] Brooks RA Di Chiro G Keller MR. Explanation of cerebral white--gray contrast in computed tomography. J Comput Assist Tomogr. (1980) 4:489–91. doi: 10.1097/00004728-198008000-00016 6771308

[32] Esdaille CJ Coppler PJ Faro JW Weisner ZM Condle JP Elmer J. Duration and clinical features of cardiac arrest predict early severe cerebral edema. Resuscitation. (2020) 153:111–8. doi: 10.1016/j.resuscitation.2020.05.049, PMID: 32590271 PMC7733324

[33] Lee BK Kim WY Shin J Oh JS Wee JH Cha KC. Prognostic value of gray matter to white matter ratio in hypoxic and non-hypoxic cardiac arrest with non-cardiac etiology. Am J Emerg Med. (2016) 34:1583–8. doi: 10.1016/j.ajem.2016.05.063, PMID: 27278721

[34] Ali A Tanirgan G Sabanci PA Sivrikoz N Abdullah T Sencer A. Relation of gray-white matter ratio with long-term cognitive functions and quality of life in patients with mild to moderate aneurysmal subarachnoid hemorrhage: a prospective observational study. Acta Neurochir. (2018) 160:181–9. doi: 10.1007/s00701-017-3374-y, PMID: 29075902

