# Supplementary material for: Grey-to-white matter ratio on computed tomography for predicting neurological outcome in patients with heat stroke: a retrospective cohort study
Source: Front Neurol. 2025 May 13;16:1556822. doi: 10.3389/fneur.2025.1556822 (PMC12106023; doi:10.3389/fneur.2025.1556822)
Supplement: Supplementary file 1 [file Data_Sheet_1.pdf]

TABLE S1 Cerebral performance categories

CPC	Disability	Conscious	Independent	Features
1	No, or minor	Yes	Yes	Able to work and lead a normal life. May have mild dysphasia, non-incapacitating hemiparesis, or minor cranial nerve abnormalities.
2	Moderate	Yes	Yes	Able to travel by public transport and work in sheltered environment. Independent in activities of daily life. May have hemiplegia, seizures, ataxia, dysarthria, or memory changes.
3	Severe	Yes	No	Limited cognition, dementia, locked-in, minimally conscious. Usually in institution, but it may be looked after at home with exceptional family effort.
4	Unconscious	No	No	Persistent vegetative state
5	Dead	-	-	Certified brain dead or dead by traditional criteria
