# Supplementary material for: Grey-to-white matter ratio on computed tomography for predicting neurological outcome in patients with heat stroke: a retrospective cohort study
Source: Front Neurol. 2025 May 13;16:1556822. doi: 10.3389/fneur.2025.1556822 (PMC12106023; doi:10.3389/fneur.2025.1556822)
Supplement: Supplementary file 2 [file Data_Sheet_2.pdf]

TABLE S2 ROC curve analysis of predicting neurological outcome of heat stroke patients in the derivation cohort

Indicators	AUC	95% CI	*p*-value	Sensitivity%	Specificity%
$GWR_{basal\ ganglia}$	0.936	0.851-1.000	＜0.0001	90.91	80.65
qSOFA	0.855	0.710-1.000	0.0005	81.82	80.65%
$GWR_{basal\ ganglia}$ +qSOFA	0.968	0.923-1.000	＜0.0001	90.91	90.32%
